# M^2^UNet: Multi-Scale Feature Acquisition and Multi-Input Edge Supplement Based on UNet for Efficient Segmentation of Breast Tumor in Ultrasound Images

**DOI:** 10.3390/diagnostics15080944

**Published:** 2025-04-08

**Authors:** Lin Pan, Mengshi Tang, Xin Chen, Zhongshi Du, Danfeng Huang, Mingjing Yang, Yijie Chen

**Affiliations:** 1 College of Physics and Information Engineering, Fuzhou University, Fuzhou 350108, China; panlin@fzu.edu.cn (L.P.); 221127186@fzu.edu.cn (M.T.); xinchen2025@yeah.net (X.C.); 2 Department of Ultrasound, Clinical Oncology School of Fujian Medical University, Fujian Cancer Hospital, Fuzhou 350014, China; duzhongshi@126.com (Z.D.); huangdf@fjzlhospital.com (D.H.)

**Keywords:** breast cancer, segmentation, multi-scale feature fusion, ultrasound image, deep learning

## Abstract

**Background/Objectives:** The morphological characteristics of breast tumors play a crucial role in the preliminary diagnosis of breast cancer. However, malignant tumors often exhibit rough, irregular edges and unclear, boundaries in ultrasound images. Additionally, variations in tumor size, location, and shape further complicate the accurate segmentation of breast tumors from ultrasound images. **Methods:** For these difficulties, this paper introduces a breast ultrasound tumor segmentation network comprising a multi-scale feature acquisition (MFA) module and a multi-input edge supplement (MES) module. The MFA module effectively incorporates dilated convolutions of various sizes in a serial-parallel fashion to capture tumor features at diverse scales. Then, the MES module is employed to enhance the output of each decoder layer by supplementing edge information. This process aims to improve the overall integrity of tumor boundaries, contributing to more refined segmentation results. **Results:** The mean Dice (mDice), Pixel Accuracy (PA), Intersection over Union (IoU), Recall, and Hausdorff Distance (HD) of this method for the publicly available breast ultrasound image (BUSI) dataset were 79.43%, 96.84%, 83.00%, 87.17%, and 19.71 mm, respectively, and for the dataset of Fujian Cancer Hospital, 90.45%, 97.55%, 90.08%, 93.72%, and 11.02 mm, respectively. In the BUSI dataset, compared to the original UNet, the Dice for malignant tumors increased by 14.59%, and the HD decreased by 17.13 mm. **Conclusions:** Our method is capable of accurately segmenting breast tumor ultrasound images, which provides very valuable edge information for subsequent diagnosis of breast cancer. The experimental results show that our method has made substantial progress in improving accuracy.

## 1. Introduction

Breast cancer has the highest incidence rate among malignant tumors in women, and its incidence has been steadily increasing and showing a trend centering on younger age groups [1]. According to the latest global cancer burden data released by the International Agency for Research on Cancer of the World Health Organization in 2024, female breast cancer has surpassed lung cancer and has become the most commonly diagnosed cancer [2]. Ultrasound imaging is a commonly used diagnostic tool in clinical practice due to its lack of radiation, ease of implementation, low cost, and wide range of applications, making it a valuable tool for assisting in the diagnosis of breast cancer [3,4].

**Figure 1:**
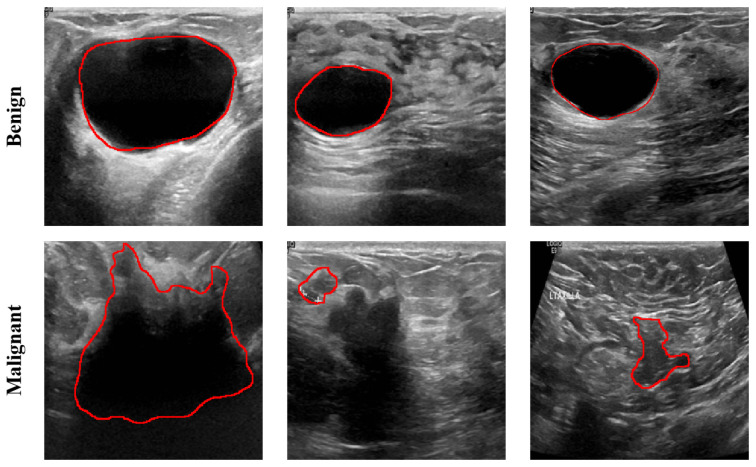
Breast ultrasound benign vs. malignant comparison image, with the red solid line denoting the boundary of the breast tumor.

Typically, factors such as the size of breast tumors, the regularity of their shape, and the presence of spiculated edges serve as criteria for the classification of benign or malignant breast tumors [5]. Malignant tumor images often exhibit characteristics, such as unclear boundaries and irregular shapes, when compared with benign tumors, as shown in Figure 1. Thus, the segmentation of malignant tumors is more challenging than that of benign tumors [6]. However, ultrasound imaging often requires the expertise of ultrasonographers for manual image acquisition and interpretation [7]. This process can be time-consuming and resource-intensive. The introduction of Computer-Aided Diagnosis (CAD) can assist ultrasonographers in ultrasound imaging and minimize subjective influences on ultrasound image interpretation [8,9]. Therefore, the development of an accurate CAD system to accurately segment breast tumors, especially malignant tumors, can provide important information about the size and shape of tumor margins for subsequent diagnosis and treatment. Breast lesion segmentation methods have been widely studied by various researchers: they are roughly categorized into traditional and deep learning-based methods.

Traditional methods often involve manual interaction, which decreases their robustness. Maolood et al. [10] proposed a fuzzy entropy segmentation method based on a level-set threshold, and it can be used to detect lesion segmentation in different types of medical images. Daoud et al. [11] decomposed breast ultrasound images (BUSIs) into superpixels to obtain the initial contour of tumors and introduced a custom graph segmentation algorithm to improve tumor contour. Kozegar et al. [12] presented a two-stage segmentation method. They initially used a novel adaptive region growing algorithm for the rough estimation of the boundary of masses. Subsequently, they used the result from the first stage as the initial contour and introduced a deformable model based on geometric edges to obtain the final segmentation results. However, this previously proposed method is not fully automatic and requires the manual marking of seed points for each mass.

In recent years, deep convolutional neural networks (CNNs) have been widely used in breast ultrasound segmentation, especially UNet [13] and its variants, which outperform traditional methods. For example, UNet++ [14] uses dense skip connections to minimize feature information loss. AttUNet [15] incorporates an attention mechanism in order to focus the network on important regions. However, this does not encode global information. TransUNet [16] replaces the encoder part of UNet with transformer, which not only can encode image features as sequences to strengthen the global context, but also can effectively utilize the low-level features. However, TransUNet often requires a large amount of data for training, and in reality, the amount of data in medical images is very limited, and when the data set is small, it is prone to overfitting phenomenon, and its segmentation effect is not ideal. DSEUNet [17] adopts deeper benchmark UNet to ensure that sufficient feature information can be obtained. However, deep nesting also means that it will lead to an increase in the amount of computation and computation time. SKUNet [18] adds a selective kernel on the basis of UNet so that the network can automatically select and blend the features from different receptive domains. However, its segmentation results, especially for malignant tumors, are often significantly different from the true values in terms of edge morphology. EHA-Net [19] preprocessed ultrasound images using a pseudocolor algorithm to enhance the contrast between tumors and their background. They then introduced a hybrid attention mechanism into the network to improve the model’s feature extraction capabilities and focus on long-range dependencies. ULS4US [20] employs a multi-input-multi-output modular phase into a two-stage segmentation method. However, cases of missed or false detections may still be present in images of small or multiple tumors.

The challenges accompanying ultrasound segmentation are mainly due to the image features of malignant tumors. Therefore, accurate segmentation of malignant tumors in ultrasound images is more challenging than the segmentation of benign tumors. To address the image characteristics of malignant tumors, such as large differences in tumor size, irregular edges, and blurred boundaries in breast ultrasound, we propose the segmentation network incorporating multi-scale features acquisition (MFA) and multi-input edge supplement (MES) modules, named as M^2^UNet. The network consists of two parts, the encoder and the decoder, with an MFA module at the bottom for the fusion of various receptive fields. Meanwhile, the MES module and deep supervision mechanism were added to the output of each layer of the decoder. This step not only complemented its segmentation results with edges but also further constrained the network to refine the segmentation findings. The contributions of this paper mainly include the following aspects:

1. This paper introduced a novel multi-scale feature acquisition module named MFA. This module adeptly harnesses semantic information from various receptive fields to facilitate the precise segmentation of tumors;
2. To address the challenges of boundary blurring and edge irregularities in breast ultrasound imaging, we have introduced a novel MES module. This module effectively mitigates these issues by complementing ultrasound tumor edges with features extracted from the decoder output;
3. We validated the effectiveness of our method by constructing a breast ultrasound dataset using all malignant tumors in collaboration with Fujian Cancer Hospital (FCBU) and performed experimental validation on this dataset and the publicly available dataset (BUSI).

## 2. Materials and Methods

### 2.1. Dataset Description

The superiority of M^2^UNet in malignant tumor segmentation was assessed by collecting ultrasound images diagnosed as malignant breast tumors at the Fujian Cancer Hospital from January 2017 to January 2021 (denoted as FCBU), and the age distribution was between 25 and 80 years old. Using the Philips iU - Elite color ultrasonic diagnostic apparatus, three hundred breast images of 150 patients were collected. The collection frequency of the ultrasonic probe was set to 5–12 MHz and the spatial resolution was 1024 × 760 pixels. The data are divided into a training set and a test set in an 8:2 ratio. During training, the training set is further split into training data and validation data in an 8:2 ratio. Since the number of images is greater than the number of patients, if the dataset is randomly divided, images of the same patient may appear in both the training set and the test set simultaneously, resulting in the leakage of training information into the test set. However, dividing proportionally by patient can ensure that the training set and the test set come from different patient groups, respectively, effectively preventing the information in the training process from having an adverse impact on the test results and ensuring the accuracy of model evaluation. Two senior ultrasound physicians with over 5 years of breast ultrasound diagnostic experience conducted post-analysis and annotation of the primary breast lesions on the ultrasound images.

The ultrasound images collected in the FCBU dataset exhibit significant diversity in tumor size, morphology, and location across different patients. Additionally, some images present blurred boundaries between tumors and surrounding tissues, posing challenges for automated segmentation. As the BUSI dataset [21] shares similar image characteristics and age distribution with the FCBU dataset and has been widely adopted for validation in numerous methodologies, we used this dataset to retrain and test the proposed network to further verify the superiority of the network. This dataset comprises breast ultrasound images of females aged between 25 and 75, collected in 2018 using both the LOGIQ E9 and the LOGIQ E9 agile ultrasound systems. The dataset encompasses 600 patients and includes 780 images with average sizes of 500 × 500 pixels. In order to correspond to the distribution of the FCBU dataset, we chose to remove multi-lesion images and normal images to ensure that all ultrasound images input into the network contain only a single tumor. Finally, a total of 630 images were obtained in the BUSI dataset for the experiment.

### 2.2. Methods

**Figure 2:**
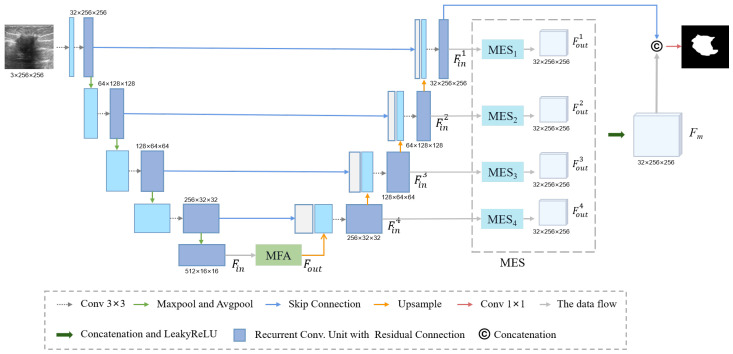
Structure of M^2^UNet.

Figure 2 shows the proposed method. The network improves the UNet used to segment breast lesions using ultrasound accurately. In M^2^UNet, initially, we incorporate residual connections into the convolutional modules of both the encoder and decoder to avoid gradient vanishing. Second, we incorporated the MFA module, which utilizes dilated convolution with different dilation rates to integrate semantic information under different receptive fields. This method can improve the adaptability of a network to multiscale targets. Finally, a multi-input edge supplement (MES_1,2,3,4_) module was used behind each decoder. This module can edge supplement the output of each decoder layer and introduce a deep supervision mechanism to refine the segmentation results further.

#### 2.2.1. Residual Block and Down-Sampling Blocks

**Figure 3:**
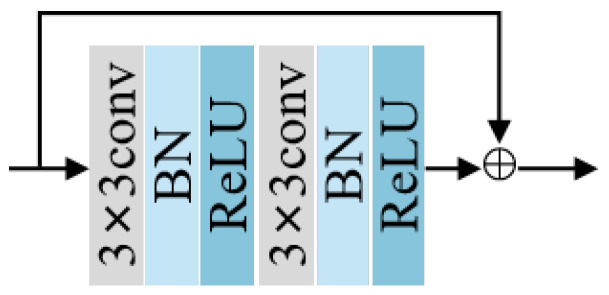
Structure of recurrent convolution unit with residual connection.

In deep learning, enhancing the network performance often involves increasing the depth of network layers. However, this process can lead to gradient disappearance. To address this issue, He et al. [22] proposed the residual module, which retains and transfers gradients effectively through skip connections. Therefore, in this paper, we consider a convolution layer, a batch normalization (BN) layer and a LeakyReLU in UNet as one unit. As shown in Figure 3, the structure containing two such units is called the recurrent convolution unit, and we add residual connections to the recurrent convolution unit.

Average-pooling and max-pooling are common pooling operations that can downscale input feature maps, reduce parameters, and enhance algorithm performance. In average-pooling, the average of pixel values within a pooling area are obtained, the sensitivity to background information is enhanced, and background retention is maximized. And max-pooling preserves the highest pixel value within a pooling area to increase the retention of texture information. In this paper, we combined average-pooling and max-pooling to preserve the background and texture information in the feature map, as shown in Equation (1).

(1) $$D=(\theta \left(\phi^{k=3}\left(\theta \left(\phi^{k=3}\left(CAT\left(Pool_{Max}\left(x\right),Pool_{Avg}\left(x\right)\right)\right)\right)\right)\right))$$

where $Pool_{Max}$ and $Pool_{Avg}$ represent max-pooling and average-pooling, respectively. CAT(·) represents the concatenation operation. The $\phi^{k=3}$ is 3×3 convolution operations, θ represents batch normalization and LeakyReLU.

#### 2.2.2. Multi-Scale Feature Acquisition Module

**Figure 4:**
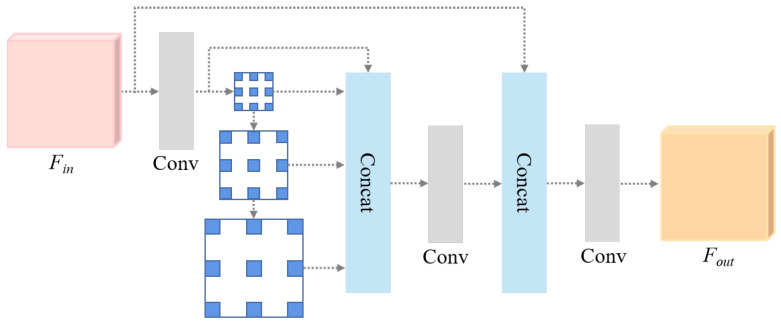
Structure of the Multi-scale Feature Acquisition (MFA) module.

Different patients exhibit various tumor sizes, locations, and morphology. As the number of network layers increases, feature information undergoes successive compression, which can lead to the loss of important information on tumors. The Spatial Pyramid Pooling (SPP) was proposed by He et al. [23] to address image distortion caused by scaling and the repetition of feature extraction in CNN. Inspired by SPP, we introduce the MFA module to extract features with different receptive fields and fuse them for multi-scale object integration, as shown in Figure 4.

The MFA module uses dilated convolution with different dilation rates to extract features of various receptive fields without increasing the amount of calculation. Suppose a dilated convolution with the same dilation rate is superimposed multiple times. In that case, the feature image will be filled with multiple redundant null values, and the semantic information of the feature itself will be lost. In addition, if the dilation rate is considerable, the characteristics of small lesions will be directly discarded. The MFA module addresses these concerns by combining dilated convolutions with four different dilation rates in a serial-parallel manner. This approach expands the receptive field while capturing multiscale contextual information. Subsequently, the original feature map $F_{in}$ is complemented with a feature map processed through dilated convolutions. This map maintains the integrity of local information, ensures feature completeness, and enhances the local spatial semantic information. Finally, a 1×1 convolution is used to reduce the dimensionality of the multiscale features, which ultimately generates the feature map $F_{out}$ for subsequent feature decoding and lesion segmentation.

The MFA module is defined in Equations (2)–(4), where CAT(·) represents the concatenation operation, ϕ shows the convolution operation, and *k* and *r* represent the convolution kernel and dilation size, respectively.

(2) $$F_{in}^{\prime }=\phi^{k=1,3,1}\left(F_{in}\right)$$

(3) $$F_{s}=CAT\left(F_{in}^{\prime },\phi_{r=2}^{k=3}\left(F_{in}^{\prime }\right),\phi_{r=4}^{k=3}\phi_{r=2}^{k=3}\left(F_{in}^{\prime }\right),\phi_{r=6}^{k=3}\phi_{r=4}^{k=3}\phi_{r=2}^{k=3}\left(F_{in}^{\prime }\right)\right)$$

(4) $$F_{out}=\phi^{k=1}\left(CAT\left(F_{in},\phi^{k=1,3}\left(F_{s}\right)\right)\right)$$

#### 2.2.3. Multi-Input Edge Supplement Module

**Figure 5:**
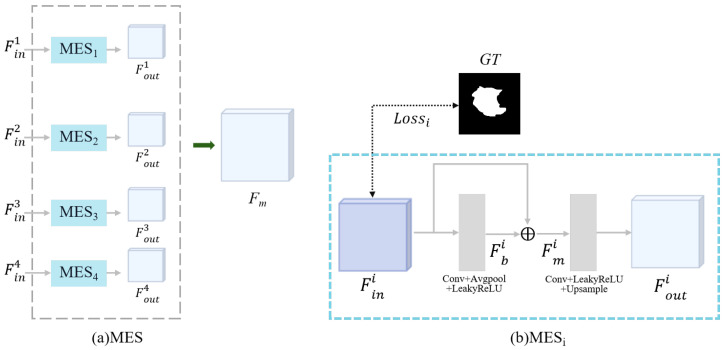
Structure of the Multi-input Edge Supplement (MES) module.

In ultrasound images, segmentation inaccuracies are mainly caused by the blurred boundaries between tumor regions and the background. The MES module is specifically designed to refine tumor edges, resulting in segmentation results that closely resemble the tumor morphology. The decoder’s output features in UNet encompass not only low-level positional information but also semantic details from the corresponding encoding layers. However, during the upsampling process, semantic information is prone to dilution, and skip connections can lead to the loss of local information. Therefore, to address this issue, we employ the MES module. As shown in Figure 5, the MES module consists of multiple $MES_{i}$ components. Specifically, the outputs from each decoder layer are progressively processed through corresponding $MES_{i}$ modules to preserve their respective detailed features. These features are aggregated and subsequently fused to enhance the final decoder layer’s output, thereby better preserving the critical edge information of tumors through this hierarchical supplementation mechanism.

The i-th MES module first obtains the local information using the 3×3 convolution and pooling layer to obtain the boundary features $F_{b}^{i}$, which effectively preserves the local information lost due to the upsampling operation. Subsequently, the feature $F_{in}^{i}$ from the i-th decoder layer is element-wise added to $F_{b}^{i}$, which yields the complemented feature map $F_{m}^{i}$. Afterward, convolution and bilinear interpolation are applied to ensure that the feature sizes are recovered to 32×256×256, which is convenient for the subsequent feature fusion. Meanwhile, to further refine the segmentation results, we adopt a deep supervision mechanism to compute the loss of $F_{in}^{i}$ with ground truth before proceeding to the MES module, as shown in Figure 5b. Through this operation, during the training process, deep semantic information can be backpropagated to shallow layers via intermediate-layer gradient paths with shorter transmission routes. Furthermore, this layer-wise deep supervision mechanism effectively mitigates information attenuation during feature transmission, enabling the network to directly propagate raw information from deep layers upward during training. This approach prevents the loss of critical information in subsequent operations and optimizes detail restoration capabilities. Finally, the outputs of each layer of the MES module are stacked through a concatenation operation to obtain the ultimate complementary results $F_{m}$. This ensures more accurate ultrasound prediction results for the network. The final output of the MES module is shown in Equations (5)–(8):

(5) $$F_{b}^{i}=L\left(Pool_{Avg}\left(\phi^{k=3}\left(F_{in}^{i}\right)\right)\right)\;i=1,2,3,4$$

(6) $$F_{m}^{i}=F_{in}^{i}⨁F_{b}^{i}\;i=1,2,3,4$$

(7) $$F_{out}^{i}=U\left(\phi^{k=1}\left(F_{m}^{i}\right)\right)\;i=1,2,3,4$$

(8) $$F_{m}=\phi^{k=3}\left(CAT\left(F_{out}^{1},F_{out}^{2},F_{out}^{3},F_{out}^{4}\right)\right)$$

where $\phi^{k}$ means the convolutional kernel of size k×k. U(·) and ⨁ represent the upsampling operation and element-wise addition. CAT(·) denotes the concatenation operation. L(·) represents the LeakyReLU.

### 2.3. Loss Function

The Cross-Entropy loss and Dice loss are used as the loss function during the training process. The input to the overall loss function consisted of four components, which correspond to the feature maps in the MES module after bilinear interpolation, and are equivalent to supervising the predicted segmentation results at each decoder stage. The loss function of M^2^UNet is shown in Equations (9) and (10).

(9) $$L_{i}=Loss_{ce}\cdot w+Loss_{dice}\cdot \left(1-w\right)$$

(10) $$Loss=\frac{\Sigma_{i=1}^{4}L_{i}}{4}$$

where $L_{i}$ denotes the loss between the layer *i* decoder prediction and the GT. The $Loss_{ce}$ and $Loss_{dice}$ represent the Cross-Entropy loss and Dice loss. Based on the grid search result, the hyperparameter *w* value is set to 0.5.

### 2.4. Evaluation Metrics

Medical image segmentation tasks are evaluated using five metrics: Dice, Pixel Accuracy(PA), Intersection over Union(IoU), Recall, and Hausdorff Distance (HD). The main operators for these are the numbers of true positives (TPs), true negatives (TNs), false positives (FPs), and false negatives (FNs) in the segmentation results. The specific calculation process for these five metrics operators is given in Equations (11)–(15).

(11) $$Dice=\frac{2TP}{2TP+FP+FN}$$

(12) $$PA=\frac{TP+TN}{TP+TN+FP+FN}$$

(13) $$IoU=\frac{TP}{TP+FP+FN}$$

(14) $$Recall=\frac{TP}{TP+FN}$$

Dice and IoU are basic metrics in segmentation tasks to evaluate the similarity between the predicted segmentation result and the true value. PA predicts the proportion of correctly predicted pixel values to the total number of pixels in the segmentation results. Recall refers to the proportion of pixels in the predicted result that are correctly segmented as foreground. High values of these indicators indicate excellent predictions.

(15) $$\begin{array}{cc} HD(X,Y) & =max\left\{\max_{x\in X}\min_{y\in Y}d\left(x,y\right),\max_{y\in Y}\min_{x\in X}d\left(x,y\right)\right\} \end{array}$$

HD is used to calculate the distance between two sets (*X* and *Y*), where a small value implies the similarity of two sets. The smaller the value of HD, the closer the edge pattern is to the ground truth.

## 3. Results

### 3.1. Experiment Setting

Our experiments were performed by using an AdamW optimizer with a batch size set to 4, the epoch set to 200, and the initial learning rate set to 0.0001. Before training, we first uniformly resize all images to 256 × 256. This is important as it makes all input images of the same size, facilitating model processing. Then, we perform random data-augmentation operations on these resized images. These operations include rotation, horizontal flip, and vertical flip, each with a probability of 0.5. These flip operations can generate different versions of the same image, effectively expanding the size of the dataset and reducing overfitting. Through these pre-processing steps, the images are better prepared for being sent to the model for training. The division ratio of the training and test sets for both datasets was 8:2. During training, the training set is further split into training data and validation data in an 8:2 ratio. All experiments were performed based on the NVIDIA GeForce RTX 3060 with 12G GPU memory.

### 3.2. Comparison Study

For the objective evaluation of the effectiveness of the M^2^UNet, several representative semantic segmentation networks were reproduced for comparison. The methods used for comparative analysis included UNet [13], TransUNet [16], ResUNet++ [24], UNet++ [14], Att UNet [15], SK UNet [18], MultiResUNet [25], DSEUNet [17] and PDF-UNet [26], and evaluations were conducted on two datasets. To ensure a fair comparison, we applied them on the same device and employed completely consistent training conditions, such as hyperparameter settings, loss functions, data partition strategies, and data augmentation approaches, to avoid performance biases. In addition, we used the paired *t*-test to calculate the *p*-value of these methods.

#### 3.2.1. Comparison with Existing Methods

**Quantitative comparisons:** As shown in Table 1, for the BUSI and the FCBU datasets, TransUNet exhibited the poorest performance among all models. This is because for specific tasks, TransUNet may require a large amount of data for training to perform optimally, and for small samples of data, it tends to lead to overfitting phenomena. Followed by Unet, this finding can be attributed to the potential information loss in UNet’s architecture and skip-connection mechanism. During feature propagation, certain details and edge information may be lost, which affects the segmentation accuracy. Variants of UNet, such as ResUNet++, UNet++, Att UNet, SK UNet, MultiResUNet, DSEUNet, and PDF-UNet, outperformed the original. Compared with the above methods, M^2^UNet demonstrates better segmentation performance in the BUSI and FCBU datasets, achieving the best results across all evaluation metrics. Specifically, M^2^UNet improves the Dice, PA, IoU, Recall, and HD on the BUSI dataset by 1.14%, 0.28%, 0.98%, 0.59%, and 2.39 mm, respectively, compared to the second-best method. Similarly, on the FCBU dataset, the corresponding improvements are 1.85%, 0.3%, 1.22%, 0.84%, and 3.83 mm. Notably, the significant enhancements in the Dice and HD are of great clinical importance for breast ultrasound tumor segmentation. In breast ultrasound images, tumor boundaries often appear blurred due to grayscale gradients and artifacts. The improvement in Dice indicates that the model’s predictions for these boundary regions are closer to the ground truth, demonstrating that M^2^UNet can more accurately identify the boundaries between tumor regions and surrounding normal tissues. Furthermore, the reduction in HD suggests that the maximum distance between the segmented boundary and the ground truth boundary has decreased, allowing for a more precise representation of the tumor’s actual shape. This further verifies the capability of our method to capture fine tumor edge details. The advancement of these two metrics demonstrates that our method can provide clinicians with more reliable diagnostic support, reducing the subjectivity of manual segmentation and thereby minimizing the risks of under-segmentation and over-segmentation. Furthermore, this lays a solid technical foundation for subsequent tasks such as benign-malignant differentiation, surgical planning, and neoadjuvant therapy evaluation.

Furthermore, we conducted experiments on benign and malignant tumors in the BUSI dataset separately, as shown in Table 2. The results indicate that, compared to the second-best PDF-UNet, M^2^UNet improves the Dice and HD by 0.46% and 1.07 mm, respectively, in benign tumor segmentation. For malignant tumor segmentation, the corresponding improvements are 2.49% and 5.07 mm. These findings demonstrate that M^2^UNet performs well in segmenting both benign and malignant tumors, with particularly significant improvements in malignant tumor segmentation. Malignant tumors typically exhibit more irregular shapes and blurrier boundaries, making their segmentation considerably more challenging than that of benign tumors. The substantial increase in Dice and the significant reduction in HD for malignant tumor segmentation further validate the effectiveness of M^2^UNet in handling issues related to boundary ambiguity and irregular tumor edges.

**Figure 6:**
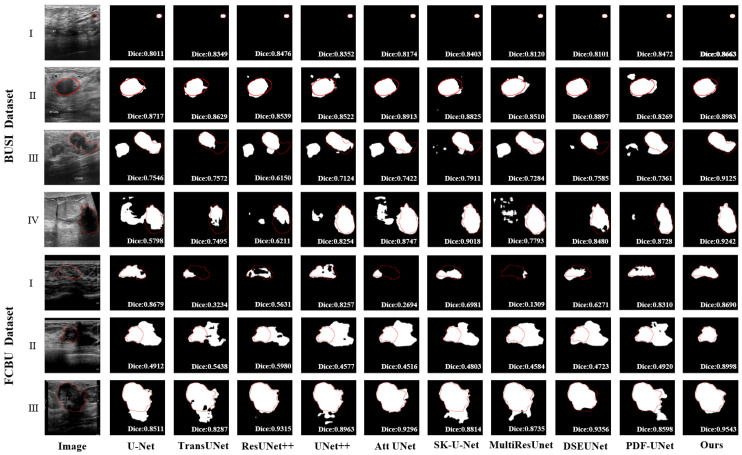
Visualization of the segmentation results of breast lesions generated by different segmentation methods. The red border is the ground truth.

**Visual comparisons:** To gain more intuitive insights into the segmentation performance, we visualized the segmentation results of each model. As shown in Figure 6, the red line is used to indicate the outline of the label. The black regions inside the red contours indicate missed segmentation areas, while the white regions outside the red contours represent extra segmentation areas. Visual comparison revealed that our method demonstrated the best segmentation results on both datasets. Specifically, compared to other methods, the segmentation results of M^2^UNet exhibit tumor shapes that more closely resemble the ground truth (GT) (Figure 6, BUSI(III)(IV), FCBU(III)). Additionally, in low-contrast images, our method effectively suppresses interference from non-tumor regions while accurately localizing the tumor. Compared to other methods, M^2^UNet significantly reduces both under-segmentation and over-segmentation (Figure 6, FCBU(I)(II)). These results indicate that M^2^UNet demonstrates significant performance advantages in tumor segmentation tasks, particularly in complex scenarios such as low contrast and ambiguous boundaries. It effectively identifies and segments tumor regions while mitigating issues related to under-segmentation and over-segmentation. The more accurate segmentation results of M^2^UNet provide a more objective and reliable basis for subsequent clinical diagnosis and treatment planning.

#### 3.2.2. Comparison with Existing Multi-Scale Modules

To validate the effectiveness of the MFA module, we conducted comparisons using several classic multiscale modules, including RFB [27], SPP [23], ASPP [28] and SPPCSPC [29]. In Table 3, although the MFA module performs slightly worse than the SPPFCSPC module in terms of PA and Recall, it achieves the best performance in Dice, IoU, and HD. The improvements in Dice and IoU indicate a more precise spatial overlap between the segmentation results and the real tumor regions, allowing for a more comprehensive capture of the overall tumor morphology. Meanwhile, the reduction in HD suggests a smaller maximum deviation between the segmented boundary and the actual tumor edge, leading to a more accurate delineation of tumor boundaries. These results reflect the effectiveness of the MFA module in tumor localization and boundary differentiation, enabling the network to produce segmentation results that are closer to the ground truth.

### 3.3. Ablation Study

To validate the effectiveness of different modules, we conducted a series of ablation experiments on two datasets. First, we replaced the convolutional modules in UNet with residual modules and combined two pooling techniques for downsampling, termed “Baseline”. Second, we added the MES module to each decoder layer, denoted as “Baseline + MES”, to assess the effectiveness of the MES module. Next, we integrated the MFA module at the bottom of the encoder in the baseline, referred to as “Baseline + MFA”, to evaluate the effect of multiscale feature fusion. Finally, we introduced the MFA and MES modules, labeled as “Baseline + MFA + MES”, to assess the combined effects of the two modules.

**Quantitative comparisons:** As shown in Table 4, compared with the baseline, the addition of the MES module resulted in improvements of 2.7% and 0.9% in the Dice scores of the two datasets and reductions of 8.9 and 1.8 mm in HD, respectively. This result indicates that supplementing tumor edges with the MES module effectively enhanced the segmentation performance. When the MFA module was added alone, the Dice scores increased by 6.36% and 1.04%, and the HD decreased by 7.22 and 1.74 mm, respectively. These results demonstrate the effectiveness of the MFA module in multi-scale feature extraction, enabling the network to segment tumor regions more accurately. The combined use of these two modules further enhances their performance, leading to an increase in Dice scores by 7.96% and 3.12% and a reduction in HD by 9.71 mm and 6.69 mm on the two datasets, respectively. Additionally, we conducted an ablation study on benign and malignant tumor segmentation in the BUSI dataset. As shown in Table 5, incorporating both the MES and MFA modules significantly improves the performance of malignant tumor segmentation, with Dice and HD increasing by 14.59% and 17.13 mm, respectively. These findings indicate that the synergistic effect of the MES and MFA modules effectively addresses challenges such as complex tumor morphology and blurred boundaries in ultrasound imaging, thereby significantly enhancing segmentation performance.

**Figure 7:**
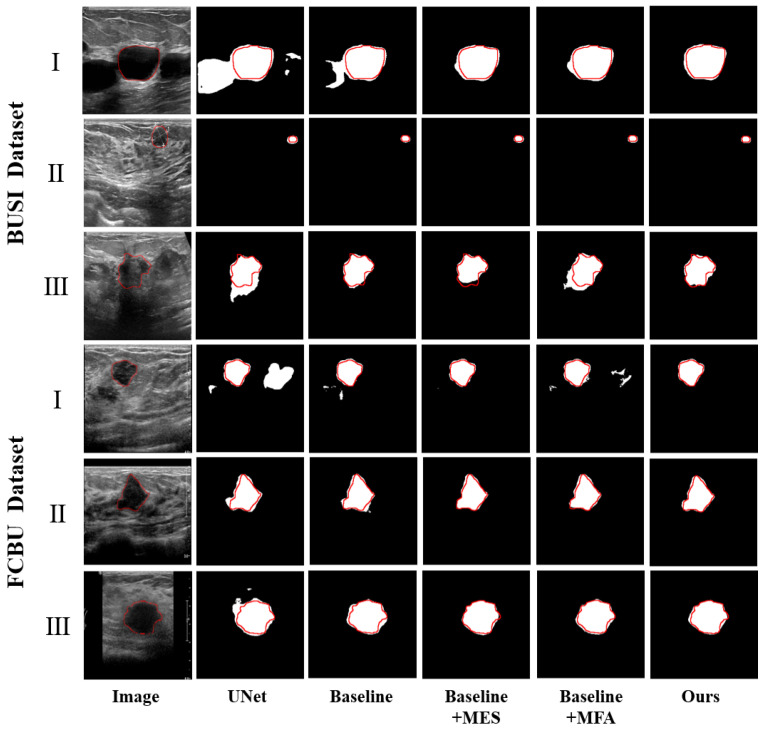
Comparison of results of ablation experiments. The red border is the ground truth.

**Visual comparisons:** Figure 7 shows the visualization results of the ablation study, where the red line is used to indicate the outline of the label. The black regions inside the red contours indicate missed segmentation areas, while the white regions outside the red contours represent extra segmentation areas. After the addition of the MES module, the segmented boundaries more closely resemble the ground truth (Figure 7, BUSI(I), FCBUII)(III)). Although introducing the MFA module may lead to over-segmentation in cases with severe noise interference (Figure 7, BUSI(III)), this issue is effectively mitigated when used in conjunction with the MES module. Overall, the inclusion of the MFA module contributes to the improvement of segmentation performance (Figure 7, BUSI(I), FCBU(II)(III)). These results indicate that multi-scale feature fusion combined with edge complementation can capture the semantic information well and refine the segmentation results effectively for both small tumors and low-contrast images.

## 4. Discussion

This paper proposes a segmentation network for breast ultrasound images, incorporating multi-scale feature acquisition modules and multi-input edge supplement modules, with the aim of assisting subsequent classification tasks. Examination of Table 2 reveals improvements in both Dice and HD for both benign and malignant tumors following the addition of the MES module and MFA module to the Baseline, respectively. Furthermore, as depicted in Figure 7, the M^2^UNet network accurately identifies and segments tumor locations, demonstrating effectiveness in handling small tumors or low-contrast images. Experimental results validate that the MES module supplements segmentation results, bringing edges closer to the GT, while the MFA module accurately segments tumors and enhances overall segmentation completeness.

In order to illustrate the efficacy of our approach in accurate segmentation results for malignant tumors, we conducted separate comparisons of existing SOTA and different modules for segmentation of malignant and benign tumors, respectively, as shown in Table 2. The data from Table 2 clearly indicate that for the segmentation of malignant tumors, the Dice coefficient of the M^2^UNet network has improved by 14.59%, while for benign tumors, it has improved by 7.49%. This observation strongly suggests that the M^2^UNet network can significantly enhance the segmentation results for malignant tumors, which is of significant importance for disease detection and clinical diagnosis. Additionally, in terms of the HD metric, the distance for malignant tumors has decreased by 17.13 mm. This also indicates that the M^2^UNet network is effective at extracting clear boundary information from low-contrast ultrasound images, thereby assisting doctors in distinguishing between benign and malignant tumors based on their edge morphology. Moreover, compared to Transformer-based methods, such as those proposed by Zhu et al. [30] and Tagnamas et al. [31], our approach achieves comparable overall performance while maintaining a more lightweight network architecture. This further demonstrates the superiority of our method.

Although M^2^UNet achieves good results in segmentation tasks, some limitations still exist. Specifically, in ultrasound images, when the noise is severe (Figure 6, BUSI(II)) or the tumor region is not prominent enough (Figure 6, FCBU(I)), the M^2^UNet model exhibits noticeable under-segmentation and over-segmentation issues. Additionally, although M^2^UNet effectively suppresses interference from non-tumor regions, when both interference and blurred edges coexist in ultrasound images (Figure 7, BUSI(III)), the model’s processing capability, is somewhat limited, resulting in less precise delineation of tumor boundary morphology. These challenges indicate directions for future work: during the data preprocessing phase, we can further employ denoising algorithms to reduce noise in images and mitigate its impact on segmentation results; introduce diverse types of ultrasound images to enhance generalization; and perform temporal feature extraction operations to capture dynamic changes of tumors between ultrasound video frames, thereby supplementing our method in the temporal dimension to improve segmentation performance. These efforts will contribute to the wider application of our method in clinical practice and provide further assistance to doctors in diagnosing and treating breast cancer.

## 5. Conclusions

In this paper, we proposed a novel breast ultrasound tumor segmentation network named M^2^UNet. This network replaces the convolutional modules with residual modules in the UNet architecture to address the issue of vanishing gradient. Subsequently, the MFA module was introduced to fuse receptive fields of different scales, which enhanced local spatial semantic information while retaining original features. In the decoder part, the MES module with a deep supervision mechanism was incorporated to supplement segmenting results with edge information, which refined the edge details. A series of experiments conducted on a publicly available dataset and a self-collected breast ultrasound dataset demonstrated that our approach can achieve more accurate breast tumor segmentation results. Furthermore, through comparison with several well-established multiscale modules, the superiority of the MFA module was demonstrated. By precisely delineating tumor boundaries, M^2^UNet assists clinicians in more intuitively analyzing tumor characteristics such as irregular margins and aspect ratios, providing objective evidence for subsequent differentiation between benign and malignant tumors and metastatic potential. This automatic segmentation method helps reduce subjective variability in human assessments, such as reducing diagnostic mistakes among less experienced clinicians. Additionally, the segmentation enables monitoring of tumor volume changes to evaluate treatment efficacy during neoadjuvant chemotherapy. These applications position the technology as crucial for early breast cancer screening and therapeutic response assessment.

## Figures and Tables

**Table 1:** The segmentation results of different competing methods on the BUSI dataset and FCBU dataset. The paired *t*-test is used to calculate the *p*-value and the best results are shown in bold.

Method	Dataset	Dice %	PA %	IoU %	Recall %	HD (mm)
UNet	BUSI	69.56 ± 2.57 ^Δ^	95.90 ± 0.43 ^*^	77.15 ± 1.31 ^Δ^	83.14 ± 2.24 ^*^	32.30 ± 3.43 ^*^
TransUNet		62.96 ± 3.07 ^Δ^	94.72 ± 0.73 ^Δ^	73.88 ± 1.62 ^Δ^	82.58 ± 2.52 ^Δ^	38.02 ± 4.11 ^Δ^
ResUNet++		70.83 ± 2.44 ^Δ^	95.91 ± 0.46 ^*^	77.73 ± 1.30 ^Δ^	85.35 ± 2.02 ^Δ^	30.71 ± 3.21 ^*^
UNet++		73.06 ± 2.23 ^Δ^	96.21 ± 0.45 ^*^	78.92 ± 1.23 ^Δ^	84.93 ± 2.04 ^*^	32.50 ± 3.40 ^*^
Att UNet		74.64 ± 2.14 ^Δ^	96.27 ± 0.45 ^*^	79.76 ± 1.17 ^Δ^	84.26 ± 2.06	32.37 ± 3.63 ^*^
SK UNet		73.19 ± 2.32 ^Δ^	96.01 ± 0.55 ^*^	79.12 ± 1.32 ^Δ^	85.23 ± 1.99 ^Δ^	30.08 ± 3.31 ^*^
MultiResUNet		73.91 ± 2.20 ^Δ^	96.12 ± 0.48 ^*^	79.36 ± 1.22 ^Δ^	84.72 ± 1.99	26.85 ± 2.93 ^*^
DSEUNet		77.06 ± 2.16	96.57 ± 0.42	81.52 ± 1.16 ^*^	85.53 ± 1.97	22.98 ± 3.02 ^*^
PDF-UNet		78.29 ± 1.92	96.56 ± 0.42	82.02 ± 1.13	86.58 ± 1.79	22.10 ± 2.55
Ours		**79.43 ± 1.97**	**96.84 ± 0.45**	**83.00 ± 1.12**	**87.17 ± 1.80**	**19.71 ± 2.67**
UNet	FCBU	86.74 ± 1.39 ^Δ^	96.95 ± 0.28 ^Δ^	87.37 ± 0.92 ^Δ^	90.84 ± 1.46 ^*^	18.63 ± 2.18 ^*^
TransUNet		84.08 ± 1.73 ^Δ^	96.29 ± 0.36 ^Δ^	85.33 ± 1.10 ^Δ^	90.38 ± 1.51 ^*^	22.37 ± 2.09 ^Δ^
ResUNet++		87.16 ± 1.13 ^Δ^	96.98 ± 0.29 ^Δ^	87.53 ± 0.89 ^Δ^	92.06 ± 1.20	18.35 ± 2.06 ^Δ^
UNet++		87.45 ± 1.07 ^Δ^	96.91 ± 0.32 ^Δ^	87.64 ± 0.86 ^Δ^	91.77 ± 1.20	19.50 ± 2.31 ^Δ^
Att UNet		88.02 ± 1.18 ^*^	97.14 ± 0.31 ^*^	88.33 ± 0.90 ^*^	92.91 ± 1.07	16.79 ± 2.00 ^*^
SK UNet		88.05 ± 0.87 ^Δ^	97.02 ± 0.28 ^Δ^	87.98 ± 0.74 ^Δ^	91.94 ± 1.22	15.39 ± 1.41 ^Δ^
MultiResUNet		87.71 ± 1.18 ^*^	96.98 ± 0.30 ^Δ^	87.91 ± 0.83 ^Δ^	92.27 ± 1.10	15.46 ± 1.70 ^*^
DSEUNet		88.71 ± 0.95 ^*^	97.10 ± 0.33 ^*^	88.63 ± 0.82 ^*^	92.46 ± 1.15	14.71 ± 1.48 ^*^
PDF-UNet		89.12 ± 1.27 ^*^	97.47 ± 0.26	89.40 ± 0.83 ^*^	93.79 ± 1.15 ^*^	14.85 ± 1.58 ^*^
Ours		**90.97 ± 0.62**	**97.77 ± 0.23**	**90.62 ± 0.59**	**94.63 ± 0.77**	**11.02 ± 1.00**

^*^ *p* < 0.05. ^Δ^ *p* < 0.001.

**Table 2:** Comparison results of benign and malignant tumors on the BUSI dataset. The paired *t*-test is used to calculate the *p*-value and the best results are shown in bold.

Methods	Benign		Malignant	
	Dice	HD	Dice	HD
UNet	72.47 ± 2.99	25.58 ± 4.07	63.80 ± 4.79	44.72 ± 5.79
TransUNet	65.19 ± 3.80	33.31 ± 5.00	58.55 ± 5.22	47.34 ± 7.04
ResUNet++	74.45 ± 2.82	25.26 ± 3.90	63.66 ± 4.50	41.74 ± 5.31
UNet++	75.14 ± 2.70	27.55 ± 4.34	68.95 ± 3.90	41.79 ± 5.19
Att UNet	74.39 ± 2.71	29.97 ± 4.79	75.13 ± 3.50	36.87 ± 5.30
SK UNet	75.98 ± 2.82	25.51 ± 4.34	67.68 ± 4.00	38.88 ± 4.68
MultiResUNet	76.10 ± 2.47	20.18 ± 3.18	69.59 ± 4.35	39.87 ± 5.54
DSEUNet	78.60 ± 2.54	19.11 ± 3.82	74.02 ± 4.00	30.80 ± 4.68
PDF-UNet	79.50 ± 2.32	16.75 ± 2.80	75.90 ± 3.41	32.66 ± 4.84
Ours	**79.96 ± 2.53**	**15.68 ± 3.26**	**78.39 ± 3.09**	**27.59 ± 4.43**

**Table 3:** Comparison results of different multi-scale modules on the BUSI dataset and FCBU dataset. The paired *t*-test is used to calculate the *p*-value, and the best results are shown in bold.

Methods	Dataset	Dice %	PA %	IoU %	Recall %	HD (mm)
Baseline + RFB	BUSI	75.65 ± 2.22	96.40 ± 0.44	80.69 ± 1.23	85.59 ± 1.97	23.10 ± 2.52
Baseline + SPP		75.79 ± 2.14	96.16 ± 0.50	80.58 ± 1.26	85.42 ± 1.99	29.17 ± 3.31
Baseline + ASPP		75.52 ± 2.24	96.38 ± 0.45	80.61 ± 1.23	85.04 ± 2.04	24.26 ± 2.88
Baseline + SPPCSPC		76.61 ± 2.12	**96.55 ± 0.44**	81.23 ± 1.19	**86.17 ± 1.88**	27.92 ± 3.45
Baseline + MFA		**77.83 ± 1.89**	96.31 ± 0.50	**81.51 ± 1.14**	85.86 ± 1.84	**22.20 ± 2.53**
Baseline + RFB	FCBU	88.58 ± 1.34	97.43 ± 0.25	89.01 ± 0.86	93.30 ± 1.19	15.00 ± 1.57
Baseline + SPP		88.34 ± 0.93	97.02 ± 0.31	88.26 ± 0.79	93.30 ± 1.04	16.73 ± 1.57
Baseline + ASPP		88.64 ± 0.82	97.19 ± 0.27	88.50 ± 0.71	92.49 ± 1.10	16.82 ± 1.68
Baseline + SPPCSPC		89.51 ± 0.90	97.26 ± 0.32	89.33 ± 0.79	**93.41 ± 1.11**	14.35 ± 1.54
Baseline + MFA		**89.74 ± 0.75**	**97.52 ± 0.23**	**89.54 ± 0.64**	93.26 ± 0.94	**14.00 ± 1.33**

**Table 4:** Ablation results on the BUSI dataset and FCBU dataset. The best results are shown in bold.

Methods	Dataset	Dice %	PA %	IoU %	Recall %	HD (mm)
UNet	BUSI	69.56 ± 2.57	95.90 ± 0.43	77.15 ± 1.31	83.14 ± 2.24	32.30 ± 3.43
Baseline		71.47 ± 2.44	96.01 ± 0.48	78.24 ± 1.32	83.77 ± 2.18	29.42 ± 3.09
Baseline + MES		74.17 ± 2.51	96.56 ± 0.47	80.36 ± 1.33	86.71 ± 2.01	20.52 ± 2.47
Baseline + MFA		77.83 ± 1.89	96.31 ± 0.50	81.51 ± 1.14	85.86 ± 1.84	22.20 ± 2.53
Baseline + MES + MFA		**79.43 ± 1.97**	**96.84 ± 0.45**	**83.00 ± 1.12**	**87.17 ± 1.80**	**19.71 ± 2.67**
UNet	FCBU	86.74 ± 1.39	96.95 ± 0.28	87.37 ± 0.92	90.84 ± 1.46	18.63 ± 2.18
Baseline		87.85 ± 1.08	97.09 ± 0.28	88.08 ± 0.85	92.26 ± 1.21	17.71 ± 1.89
Baseline + MES		88.75 ± 0.97	97.21 ± 0.28	88.76 ± 0.81	93.02 ± 1.14	15.91 ± 1.77
Baseline + MFA		88.89 ± 0.90	97.36 ± 0.25	88.86 ± 0.71	93.09 ± 0.98	15.97 ± 1.74
Baseline + MES + MFA		**90.97 ± 0.62**	**97.77 ± 0.23**	**90.62 ± 0.59**	**94.63 ± 0.77**	**11.02 ± 1.00**

**Table 5:** Ablation results of benign and malignant tumors on the BUSI dataset. The best results are shown in bold.

Methods	Benign		Malignant	
	Dice	HD	Dice	HD
UNet	72.47 ± 2.99	25.58 ± 4.07	63.80 ± 4.79	44.72 ± 5.79
Baseline	74.25 ± 2.88	24.52 ± 3.66	65.96 ± 4.43	39.33 ± 5.40
Baseline + MES	75.79 ± 3.08	**14.53 ± 2.50**	70.96 ± 4.32	32.06 ± 4.92
Baseline + MFA	79.21 ± 2.29	16.70 ± 2.78	75.10 ± 3.33	32.94 ± 4.75
Baseline + MES + MFA	**79.96 ± 2.53**	15.68 ± 3.26	**78.39 ± 3.09**	**27.59 ± 4.43**

## Data Availability

The BUSI dataset used in this study can obtain from the following url: https://scholar.cu.edu.eg/?q=afahmy/pages/dataset, accessed on 6 April 2024. The FCBU dataset is available from the corresponding authors.
